# Raman Spectroscopy detects changes in Bone Mineral Quality and Collagen Cross-linkage in Staphylococcus Infected Human Bone

**DOI:** 10.1038/s41598-018-27752-z

**Published:** 2018-06-20

**Authors:** Mohamed Khalid, Tanujjal Bora, Ahmed Al Ghaithi, Sharanjit Thukral, Joydeep Dutta

**Affiliations:** 1 Department of Orthopaedics, College of Medicine, Taibah University, Universities Road, Taibah, Madinah Al-Munawwarah, 42353 Saudi Arabia; 2 Centre of Excellence in Nanotechnology, Asian Institute of Technology, PO Box 4, Klong Luang Pathumthani, 12120 Thailand; 3 Oman Medical Specialty Board, Orthopaedic Residency Program, Al-Khoud, Al-Athiba Oman; 4 Microbiology Department, College of Medicine, Sultan Qaboos University, Al-Khoud, 123 Oman; 5 Functional Materials, Department of Applied Physics, SCI School, KTH Royal Institute of Technology, SE-164 40, Kista, Stockholm Sweden

## Abstract

Diagnosis of osteomyelitis presents a formidable challenge. Lack of pathognomonic clinical sign(s) and diagnostic tests that can diagnose osteomyelitis at an early stage contribute to this difficulty. If the diagnosis is not made early, the disease becomes very difficult to eradicate and can lead to limb threatening and potentially life-threatening complications. *Staphylococcus aureus* is the most common organism causing osteomyelitis. Raman Spectroscopy provides information about molecular vibration that could potentially be harnessed as a spectral signature for cellular changes in specific pathologic conditions. In this study we describe a technique using Raman spectroscopy that could potentially be used to diagnose staphylococcal osteomyelitis. Human bone samples were co-cultured with *Staphylococcus aureus* (*S. aureus*) and the effects of bacterial growth on bone quality were then monitored using Raman spectroscopy. A major drop in the bone mineral quality and crystallinity was observed in the infected bones compared to the controls. *S. aureus* infection was also found to alter the collagen cross-linking. Our study shows that specific spectral signatures are present for the cause as well as the effect of staphylococcal osteomyelitis, opening the possibility of developing a useful diagnostic modality for early and rapid diagnosis of this condition.

## Introduction

Osteomyelitis is an inflammatory process accompanied by bone destruction caused by an infecting micro-organism^1^. It is often a challenging condition for the patient as well as the treating physician. For the patient it could mean severe and un-relenting pain, temporary, or at times, permanent loss of mobility, with or without systemic symptoms, such as fever and chills. For the physician, lack of pathognomonic signs, as well as, specific diagnostic tests that can detect the condition early can prove to be frustrating^2^.

Pathogenesis of osteomyelitis involves the agent (micro-organism), the host, and the environment^3^. The focus of this work is on the specific interaction between the bone and *Staphylococcus aureus*, which is the predominant organism causing osteomyelitis^4–7^. *Staphylococcus aureus* (*S. aureus*) secretes a myriad of extracellular substances and cell associated factors that contribute to its virulence.

*Staphylococcus aureus* also has the ability to invade mammalian cells and this may explain its capacity to colonize tissues and to persist after bacteremia^8^. It has been reported that *S. aureus* that has been internalized by cultured osteoblasts can survive within the cells^9^. This intracellular survival (at times in a metabolically altered state in which they appear as so-called small-colony variants) could explain the persistence of bone infections^10^. Isolation of the offending micro-organism by blood cultures or direct cultures remain the cornerstone for the management of osteomyelitis^11,12^, as targeted antibiotics can then be administered. Blood cultures are positive only in hematogenous acute osteomyelitis cases and direct cultures involve a surgical procedure with its attendant anesthetic and surgical risks. Thus, a non-invasive way of obtaining a specific diagnosis would be of immense value. In this study we have performed some preliminary *in vitro* experiments using Raman spectroscopy to explore this modality as a potential technique for rapid diagnosis of staphylococcal osteomyelitis.

Raman spectroscopy is based on inelastic scattering of monochromatic laser light providing useful vibrational information of chemical bonds and symmetry of molecules^13^. Over the last decade several investigators have reported the usefulness of Raman spectroscopy in the diagnosis of a variety of bone diseases in humans, that has been summarized chronologically as a table in the supplementary information^14–22^. These reports range from studies looking at mineralization and structural deterioration of bone in osteoporosis^14,20^, structural deterioration of collagen of nails in osteoporosios^22^, monitoring treatment of osteoporosis^15,17^, and monitoring a major adverse reaction to the treatment of osteoporosis such as osteonecrosis of jaw^19^. Other diseases affecting the structure of bone such as osteomalacia^18^, and osteogenesis imperfecta^21,22^ have also been studied using Raman spectroscopy. Of particular relevance to this study, is the work of Esmond-White *et al*.^16^, who showed that diabetic osteomyelitis patients requiring surgical intervention had abnormal calcium phosphate minerals including dicalcium phosphate dihydrate (brushite) and uncarbonated apatite. In this work normal human bone samples were collected from patients undergoing knee replacement surgery and *S. aureus* infection was introduced *in-vitro* into the samples. The bacterial growth and its effects on the bone quality were then systematically monitored using Raman spectroscopy over a period of three weeks.

## Materials and Methods

### Sample preparation

The study was approved by Sultan Qaboos University medical ethics committee. The methods were carried out *in accordance with* the relevant guidelines and regulations of the ethics committee. Bone samples were obtained in an aseptic manner from four patients undergoing total knee arthroplasty after obtaining informed consent. Each bone section was divided into four quadrants and five cancellous bone pieces measuring 2-3 mm in diameter were cut from each quadrant and kept moist in swabs soaked in sterile normal saline (0.9% NaCl solution, pH 7.4). Thus, a total of 80 samples from four patients were obtained. A bacterial inoculum of *S. aureus* was created from a fresh overnight culture which was subsequently diluted with 10 ml sterile saline water to a concentration within the range of 1 × 10^6^ to 1 × 10^7^ colony forming units (CFU)/ml, and the bone pieces were added to the mixture. Control bone samples were then collected in a sterile container containing 10 ml sterile normal saline water. Bone pieces (both inoculated and control samples) were then incubated for 21 days at 37 °C to facilitate bacterial growth. Samples from the control specimens were cultured every 48 hours to ensure that they remained sterile.

### Raman measurement

Raman spectra were recorded using a confocal Raman microscope (XploRA from HORIBA Jobin Yvon, France) fitted with 25 mW 532 nm laser (laser spot size: 2 μm). A 1% transmittance filter was fixed at the probe laser station to avoid sample heating by the laser. An integration time of 20 s was fixed for all Raman measurements and spectra were collected with a 10 × objective lens over a spectral range of 200 to 2000 cm^−1^ using a TE cooled CCD camera (Syncerity, HORIBA, France) attached to a monochromator of the spectrometer with 1800 gr/mm grating providing spectral resolution better than 2 cm^−1^. The growth of *S. aureus* and its effect on bone quality was monitored for 21 days. For Raman measurements we have taken one bone piece at a time and collected signals from 5 different spots from the same sample.

### Data analysis and representation

Raman spectra obtained from each sample were first baseline corrected by using LabSpec 6 software for background signal correction and averaged to obtain low noise signal. Then all the averaged Raman spectra were normalized with respect to the height of the CH_2_ wag Raman band at 1450 cm^−1^ for further analysis. Selection of this band (1450 cm^−1^) was done based on minimal changes observed in the data. For that we have carefully scanned all the spectra and found minimal spectral changes in the 1450 cm^−1^ peak and therefore fixed the normalization point around this peak. Although *S. aureus* has a Raman band at 1465 cm^−1^, the intensity and width of the CH_2_ wag Raman band at 1450 cm^−1^ are much higher than the Raman band from bacteria and hence no considerable influence of Raman signal from the bacteria could be observed.

The data obtained after normalization was then represented as follows:

$$\hat{a}\pm s$$

where $\hat{a}$ is the mean intensity/area of the peak of interest and $s$ is the standard deviation of measurement.

$\hat{a}$ was calculated by equation (1):

1 $$\hat{a}=\frac{1}{n}\sum _{i=1}^{n}{a}_{i}$$

where $n$ is the number of samples and ${a}_{i}$ is the intensity/area of the *i*^th^ Raman peak.

Standard deviation of measurement ($s$) was then calculated using equation (2),

2 $$s=\sqrt{\frac{1}{n-1}\sum _{i=1}^{n}{(\hat{a}-{a}_{i})}^{2}}$$

### Data availability

The datasets generated during and/or analyzed during the current study are available from the corresponding author on reasonable request.

## Results

**Figure 1:**
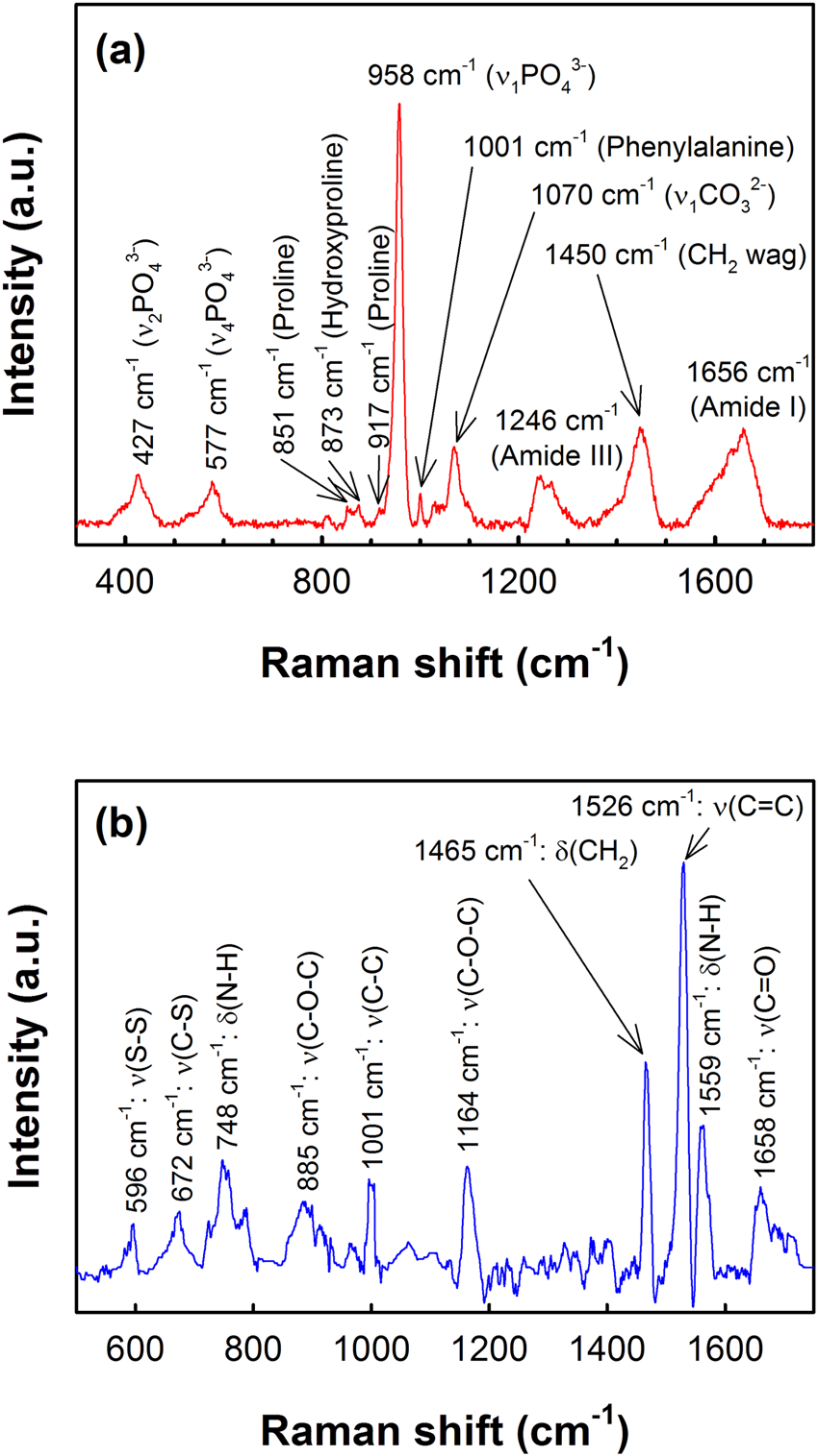
Typical Raman spectrum of (**a**) healthy human bone and (**b**) *S. aureus* bacteria obtained with 532 nm laser excitation. Spectra were background corrected for clarity of presentation. (ν: stretching mode, δ: deformation mode).

Figure 1(a) shows a typical Raman spectrum of healthy human bone, along with the assignments of the Raman bands. Raman bands at 427, 577 and 958 cm^−1^ are characteristics of the vibrational modes of phosphate, while the Raman band associated to carbonate bone mineral is located at 1070 cm^−1^ ^24^. Peaks at 851, 873 and 917 cm^−1^ are assigned to the collagen proline and hydroxyproline matrix and the peak at 1001 cm^−1^ is characteristic of phenylalanine. Protein related amide III Raman band is located between 1210 to 1320 cm^−1^, typically consisting of two major components at 1246 cm^−1^ and 1270 cm^−1^ assigned to the collagen *β*-sheet and *α*-helix secondary structures, respectively^25,26^. The peak at 1450 cm^−1^ is usually due to the CH_2_ deformation (wagging) of protein. The broad Raman band centered at 1656 cm^−1^ represents the amide I of collagen, which typically consist of several secondary structures^27^. The Raman spectrum of *S. aureus* bacterial suspension in water is shown in Fig. 1(b). The characteristic Raman band of the carotenoid pigment of *S. aureus* can be observed at 1526 cm^−1^ ^28^.

### Alterations in collagen network

**Figure 2:**
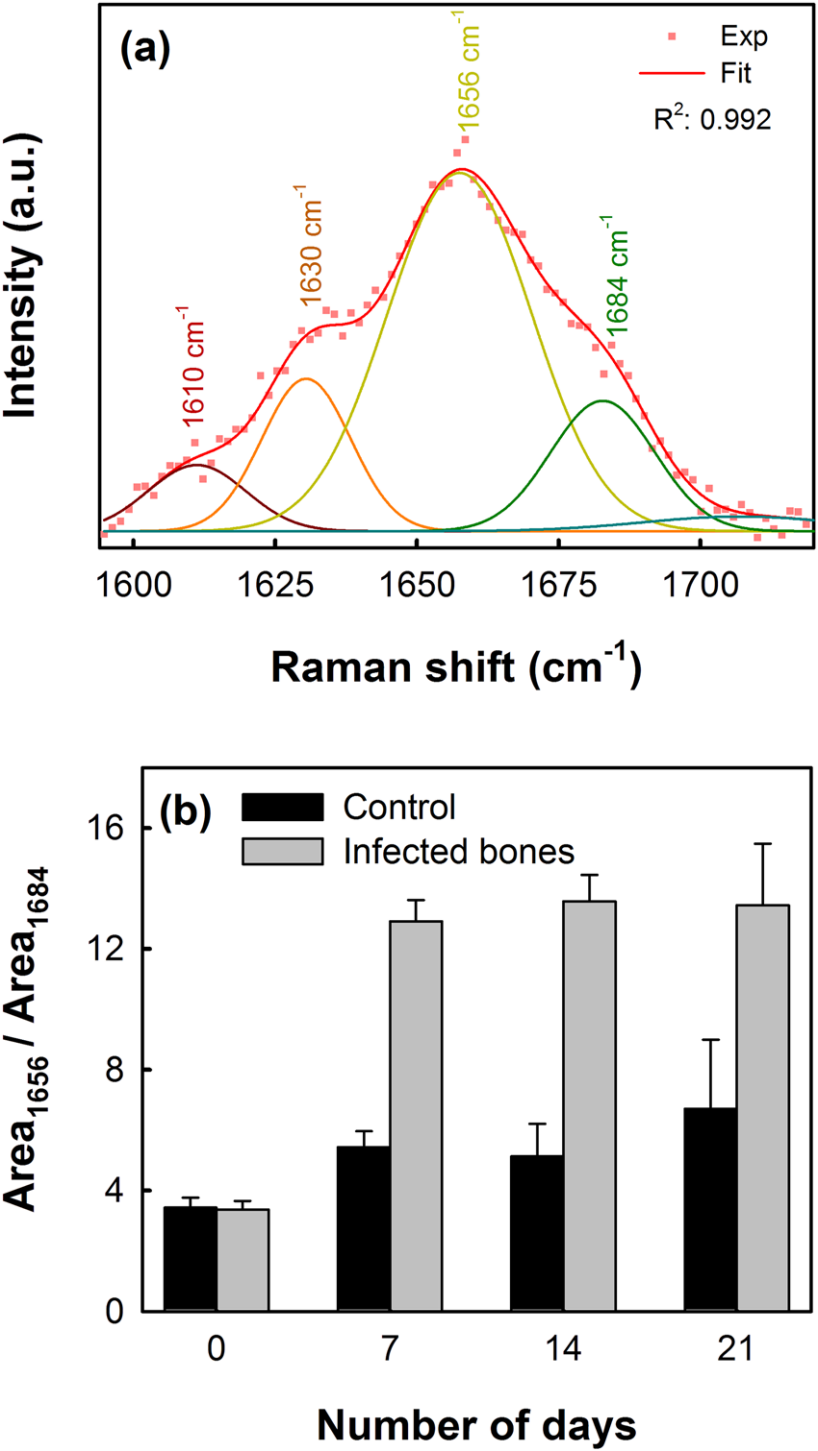
(**a**) Typical curve fitting of Amide I Raman band centered at 1656 cm^−1^ showing the center of the secondary structures of the amide I band and (**b**) collagen crosslinking ratio shown as the area ratio of Raman bands at 1656 cm^−1^ to 1684 cm^−1^ representing the non-reducible and reducible collagen types respectively.

To investigate the spectral changes in the secondary structures of collagen upon bacterial infection, curve fitting procedure was carried out to partially resolve the amide I band, which showed four major secondary bands centered at 1610, 1630, 1656 and 1684 cm^−1^, as shown in Fig. 2(a).

The collagen quality parameter can be assessed from the ratio of area under the 1656 cm^–1^ to 1684 cm^–1^ secondary structures in the amide I spectral region, which refers to the non-reducible (trivalent) to reducible (divalent) collagen crosslinking ratio^13,24^. Figure 2(b) shows the non-reducible/reducible collagen crosslinking ratio of bone samples with and without (control) bacterial infections. A considerable increase in the collagen crosslinking ratio (from 3.38 ± 0.27 to 12.91 ± 0.71), compared to the control samples (from 3.41 ± 0.31 to 5.43 ± 0.47), was observed within the first week for the *S. aureus* infected bones, representing an increase in the amount of the trivalent non-reducible cross-links due to the transformation of the divalent reducible and/or subsequently from the decrease of the reducible collagen type or its reduced formation. Between the second and third week no substantial changes were observed in the crosslinking ratio. A student t-test carried out between the two set of data returned a *p*-value of 0.052.

**Figure 3:**
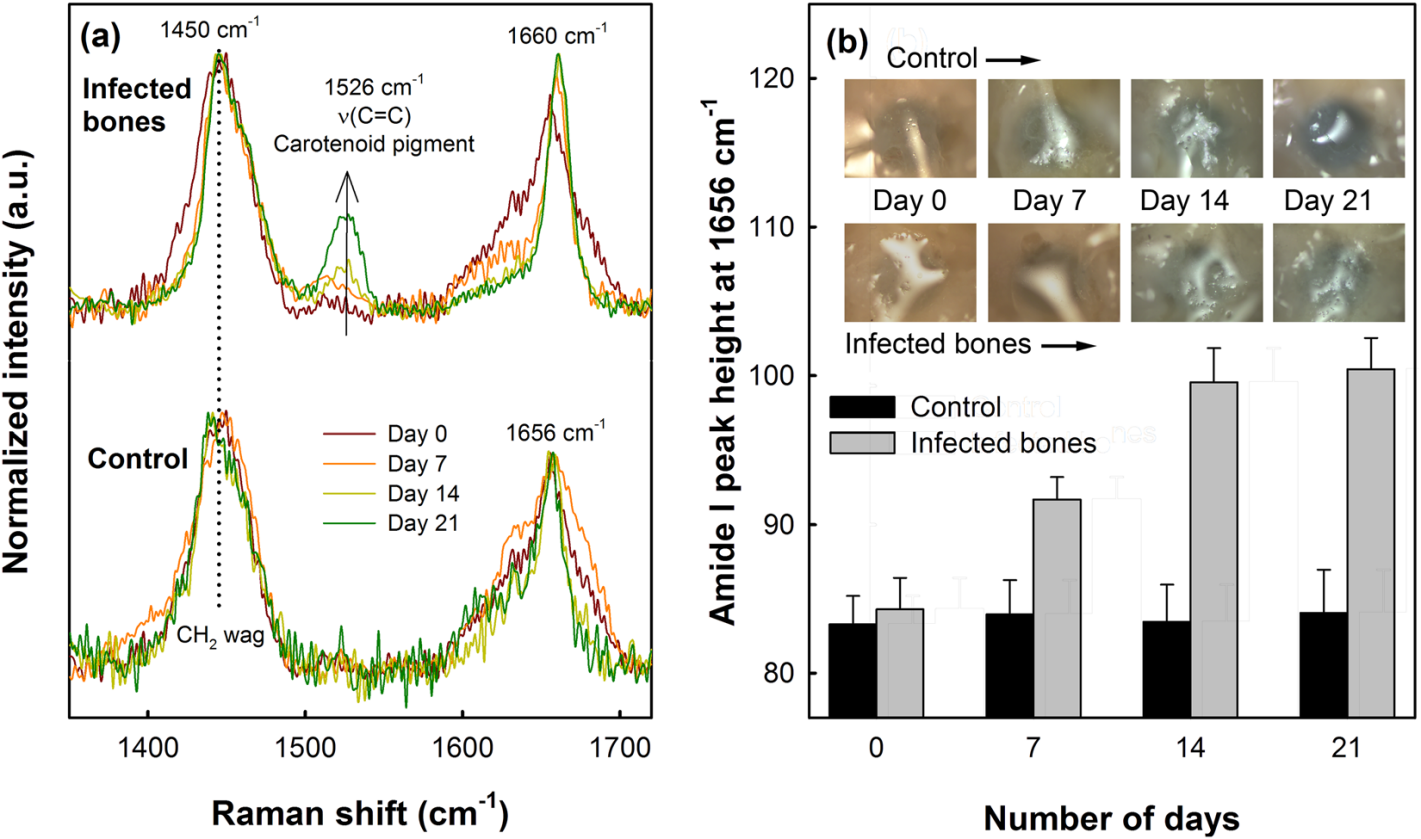
(**a**) Variations in amide I Raman bands of the control (down) and *S. aureus* infected bones (above) up to 21 days. For comparison, spectra were normalized to the CH_2_ wag band at 1450 cm^−1^. (**b**) Variations in the Amide I band intensity at 1656 cm^−1^ for the control and infected bones with respect to the number of incubation days. Inset shows the optical images (size: 500 μm × 500 μm) of control and infected bone surfaces showing no considerable visual changes in the samples during the incubation periods.

The intensity of the amide I band of infected bones at 1656 cm^−1^ was also compared to control samples for the evaluation of altered collagen quality, as shown in Fig. 3. In the case of the control sample, no significant variations in the Amide I Raman band were observed during the 21 days of incubation. However, the height of the Amide I band for the *S. aureus* infected bones was found to increase continuously over the period studied here. A shift of the amide I band from 1656 cm^−1^ to 1660 cm^−1^ in the case of the bacteria infected bones was also observed, along with the characteristic Raman band of carotenoid pigment from *S. aureus* bacteria at 1526 cm^−1^, which can be observed to increase continuously in the infected bones as the incubation period was increased indicating the bacterial cell growth. Whereas the carotenoid Raman band, as expected, was not detected in the control samples or in the absence of *S. aureus* bacteria. Although we obtained a low *p*-value (0.054) from t-test, visually no changes could be observed between the control and the infected bones during the incubation period as shown in the inset of Fig. 3(b).

### Mineral-to-Matrix ratio

The parameter *mineral-to-matrix ratio* (MMR), which is a measure of the amount of mineralization in bone, provides information on bone tissue compositions^29^. MMR is typically calculated as the ratio of mineral specific Raman band intensities (phosphate and carbonate bands at 958 and 1070 cm^−1^, respectively) to the intensity of amide I band at 1656 cm^−1^ or as a ratio of phosphate band intensity to the total intensity of proline and hydroxyproline Raman bands at 851 cm^−1^, 873 cm^−1^ and 917 cm^−1^. MMR calculated using amide I band is not solely specific to the collagen matrix, while the latter, estimated from proline and hydroxyproline is specific to the changes in the collagen matrix.

**Figure 4:**
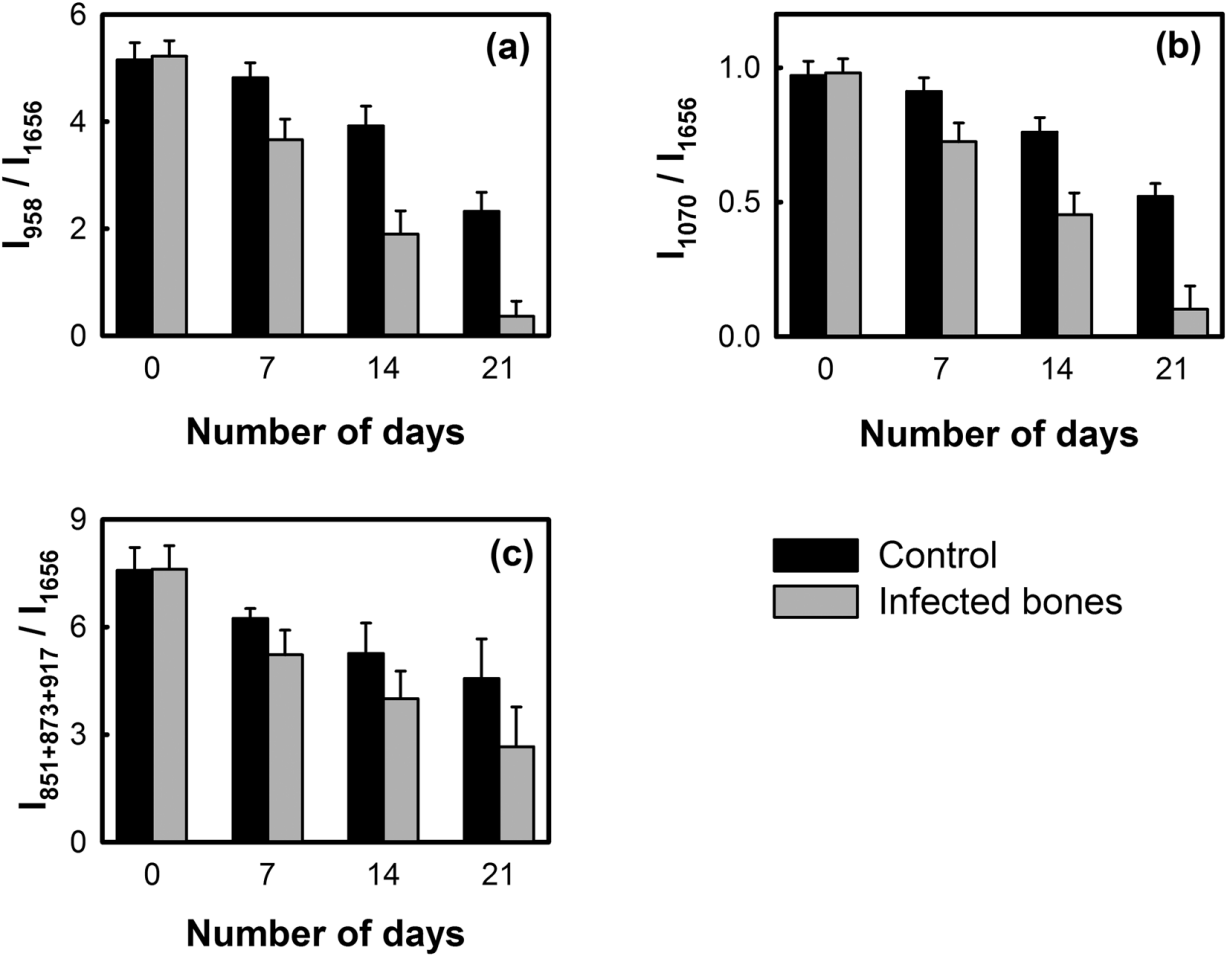
Mineral-to-matrix ratio (MMR) of control and infected bone samples estimated from the intensity ratio of (**a**) primary phosphate band at 958 cm^−1^, (**b**) carbonate band at 1070 cm^−1^ and (**c**) total intensities of proline and hydroxyproline bands at 851 cm^−1^, 873 cm^−1^ and 917 cm^−1^ to the intensity of amide I band at 1656 cm^−1^.

Figure 4 shows the MMR of human bones with and without *S. aureus* infections estimated by using both amide I and proline/hydroxyproline bands. In case of the healthy bones (control samples), mean phosphate/amide I ratio (Fig. 4(a)) was found to decrease gradually with time from 5.15 ± 0.32 to 2.33 ± 0.35 during the 21 days of incubation period. However, a substantial decrease in the mean phosphate/amide I ratio was found in the case of *S. aureus* infected bones showing almost 93% reductions in phosphate/amide I ratio during the incubation period yielding a final ratio as low as 0.36 ± 0.28 (*p*-value: 0.063). Similarly, the carbonate/amide I (Fig. 4(b)) ratio also reduced by 90% (from 0.98 ± 0.04 to 0.10 ± 0.08) upon the incubation with *S. aureus* within 21 days, compared to 47% reduction (from 0.97 ± 0.05 to 0.52 ± 0.04) observed in the absence of any bacteria (*p*-value: 0.058).

The MMR calculated by using proline/hydroxyproline Raman bands is shown in Fig. 4(c), which also indicated faster decay in the amount of mineralization in the case of the *S. aureus* infected bones compared to the healthy bones. In case of the healthy bones, almost 40% reduction in the mean MMR was observed (from 7.58 ± 0.63 to 4.56 ± 1.10) within 21 days, whereas in infected bones the ratio decreased to 2.64 ± 1.11 (*p*-value: 0.084) in 21 days which is almost 65% lower than the initial value.

### Mineral quality and crystallinity

Mineral quality of infected bones was then studied by comparing the area of carbonate-to-phosphate band ratio, which provides information on the quality of minerals or chemical composition of bones that varies with bone architecture, age and mineral crystallinity^30,31^.

**Figure 5:**
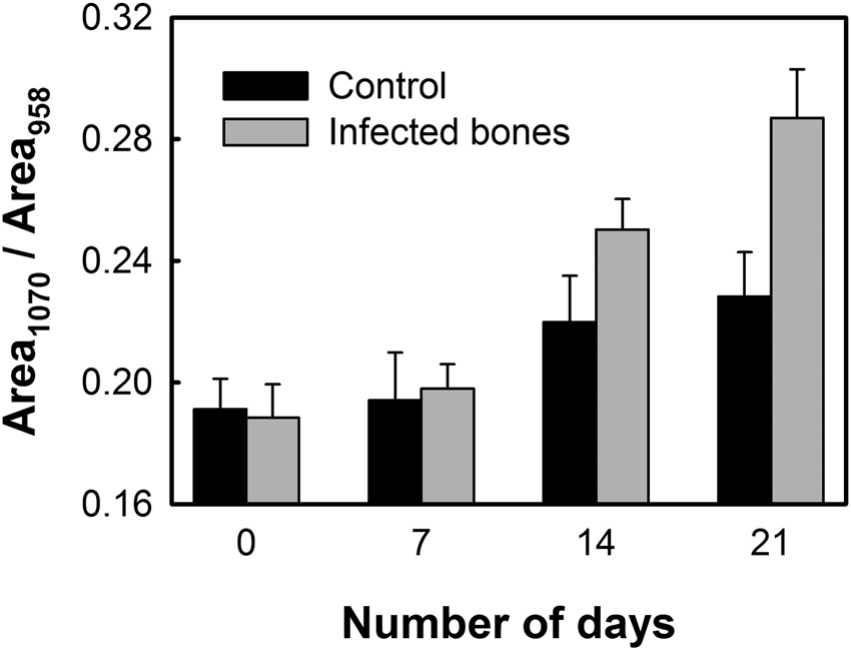
Carbonate-to-phosphate ratio of the control and infected bone samples estimated from the area ratio of the carbonate band at 1070 cm^−1^ to the phosphate band at 958 cm^−1^.

Figure 5 shows the carbonate-to-phosphate band ratio for the healthy and *S. aureus* infected bones. Within the first week of incubation, healthy or infected bone samples did not show any significant changes in the carbonate-to-phosphate ratio. However, as the incubation period was increased, the carbonate-to-phosphate ratio was observed to increase at a higher rate in the case of the infected bones compared to the healthy bones. At the end of the third week, almost 53% increase in the ratio (from 0.188 ± 0.011 to 0.287 ± 0.016) was recorded for the *S. aureus* infected bones, whereas the bones incubated in the absence of *S. aureus* showed only about 22% increase (from 0.191 ± 0.010 to 0.228 ± 0.014) after 21 days (*p*-value: 0.078).

**Figure 6:**
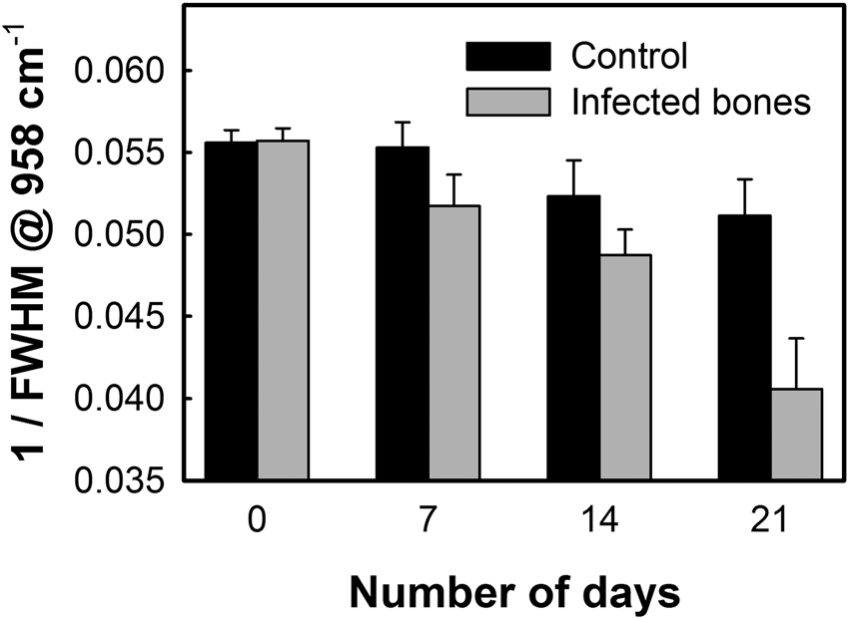
Mineral crystallinity in the control and infected bones estimated as the reciprocal of the full-width-half-maximum (FWHM) of the phosphate band at 958 cm^−1^.

From Raman spectrum of bone, mineral crystallinity can be estimated by using the width of the primary phosphate band, which is mathematically described as the reciprocal of the full-width-half-maximum (FWHM) of the phosphate band located at 958 cm^−1,^^32^. A narrower FWHM of the phosphate band will, therefore, represent higher degree of mineral crystallinity, and vice versa. Figure 6 shows the variations of mineral crystallinity in the bones with and without the *S. aureus* infections, estimated from the FWHM of the phosphate band at 958 cm^−1^. In both the cases the mineral crystallinity was found to decrease, while compared to the healthy bones, the decay rate of crystallinity was much faster in the infected bones. After three weeks of incubation, the mineral crystallinity of the infected bones dropped to 26% of the initial value compared to the 9% drop observed in the case of the healthy bones indicating higher loss of mineral crystallinity upon bacterial infections (*p*-value: 0.073).

## Discussion

Osteomyelitis imposes a great challenge for the treating physician. Despite significant clinical and socioeconomic burden, existing resources to diagnose osteomyelitis at an early stage remain inadequate. The current practice depends on the clinical examination, laboratory results and radiological features which are regarded as non-conclusive in most cases^33–35^. In this study we have systematically investigated the changes that *S. aureus* bacterial infection (*in-vitro*) produces in human bone samples using Raman spectroscopy. Ability to make measurements in the back-scattering mode, without the need for the light to pass through the tissue is a major advantage as it allows *in-vivo* analysis of tissues. The purpose of this study was to investigate the bone quality upon *S. aureus* infection, which is the predominant microbe causing osteomyelitis.

The human bone samples co-cultured with *S. aureus* bacteria showed a significant loss in the bone quality and protein conformation over time, which were studied *in-vitro* and compared with control samples (without bacterial infections). The intermolecular crosslinking of bone collagen is a key element contributing to robust fibrillar scaffolds with important mechanical properties such as tensile strength and viscoelasticity^36^. The amide I Raman band is typically an indicator of protein conformation due to the role of the amide moiety in cross linking and bonding^37^. Alterations in collagen network due to the staphylococcal infection, leading to weaker bone strength, can be analyzed from the secondary structures of collagen obtained by partially resolving the broad amide I Raman band at 1656 cm^−1^, as shown in Fig. 2(a). The non-reducible to reducible collagen crosslinking ratio (Fig. 2(b)) show that the quantity of trivalent non-reducible collagen cross-links increases significantly in case of the *S. aureus* infected bones compared to the control samples. The type I collagen, which is synthesized by osteoblasts, undergoes extensive posttranslational modifications leading to a characteristic pattern of cross-links that defines the structural and mechanical properties of bones, and hence any disorder will result in dysfunction of bone tissue. Increasing of the non-reducible collagen crosslinking at a faster rate, therefore, implies that the collagen fibers get more strongly interconnected upon bacterial infection losing their elastic property^38^. Variations in the collagen quality were also evident from the increasing height of the amide I Raman band with increasing incubation time, observed in case of the *S. aureus* infected bone samples (Fig. 3). An increase in the amide I band height at 1656 cm^−1^ has been attributed to altered collagen quality induced by various factors such as aging^9^, dehydration^10^ and radiologic damages^11^. However, these factors can be ruled out from influencing our results, since all samples used in this study were collected from patients of similar ages, kept moist during incubation period with sterile saline water and no radiological conditions were applied in the study. The observed increase in the amide I peak height can be correlated to the increase in the non-reducible collagen types as observed earlier in Fig. 2(b). A shift of the amide I band from 1656 cm^−1^ to 1660 cm^−1^ in the case of the infected bones (Fig. 3(a)) also indicates raptured collagen crosslinking^12^.

The quality and strength of bone is not only dependent on collagen crosslinking, but also on the amount of mineralization, mineral quality and crystallinity. The amount of mineralization estimated as *mineral-to-matrix ratio* (MMR) clearly indicate that upon *S. aureus* invasion, the loss of relative mineral content in bones is higher compared to the healthy bones (Fig. 4). This affects the bone strength by making them weaker at a faster rate rendering them susceptible to fracture^33,34^. The characteristic carbonate band around 1070 cm^−1^ in the Raman spectrum (Fig. 1(a)) also indicates phosphate positions in the apatitic lattice that are prone to ionic substitution, known as “B-type” carbonate substitution^24,39^. It has been reported in mouse model study that stiffness and bending modulus of bone depend significantly on the degree of mineralization, mineral crystallinity, and B-type carbonate substitution^40^. We have found almost 22% increase in the carbonate-to-phosphate ratio (Fig. 5) of the control bone samples in 21 days, whereas the ratio was found to further increase by almost 53% in the case of the *S. aureus* infected bones. This suggests that the B-type carbonate substitution is enhanced upon bacterial infection leading to an increase in the brittleness of the bones^41^.

The ability of bone to perform its mechanical functions is also strongly dependent on its mineral crystallinity, which reflects the changes in the mineral crystal size and lattice perfections^42,43^. Variations in the mineral crystallinity, and thereby alterations in crystal dimensions affects bone mechanics by inducing micro-strains within and around the crystal lattice. In human cortical bone, it has been reported that the tissue-level strength and stiffness increase with increasing crystallinity while the ductility reduces^44^. In the case of the *S. aureus* infected bone samples, we found almost 26% reduction in the crystallinity within 21 days of infection which was more than twice of the control bone samples. Therefore, the increased B-type carbonate substitution ratio and reduced crystallinity found upon *S. aureus* infection strongly implies a severe loss in the bone mineral quality and mechanical strength, which can lead to possible bone death or make them prone to fracture. However, it is also important to mention here that especially for the samples infected with bacteria after prolonged incubation, that the resultant Raman signals are also influenced by increasing bacterial concentration on the sample surface. To conclude any bacteria induced changes in the bone samples, especially the infected bone samples after a period of incubation, multiple analysis from different region is necessary since interference from bacteria during the Raman measurements cannot be avoided. This will be more important in the case of *in-vivo* studies where spectral contributions from the bacteria during Raman measurements cannot be removed and hence this *in-vitro* study is closer to actual conditions that will be faced during real-time *in-vivo* measurements.

In summary, we have used Raman spectroscopy to investigate the adverse effect of *S. aureus* infection on human bone samples and showed possible use of the technique as an early diagnostic tool for staphylococcal osteomyelitis. The implications of early diagnosis, especially if achieved non-invasively would be immense. It would facilitate early specific treatment of the patient and help rule out other conditions such as avascular necrosis. In this work we have focused on the interactions between the bone and bacteria without seeking to understand the possible effects of immune response, which might alter the bacterial degradation profile. This can be further followed separately in an *in-vivo* study.

One of the main draw-backs of Raman spectroscopy, that so far has partly limited the translation into the clinic, is the relatively low efficiency of the inelastic light scattering compared to elastic scattering, fluorescence emission or absorption of infrared light^45^. The clinical implication of this limitation is that it makes *in-vivo* imaging of deeper structures such as bones difficult. We are currently exploring two different approaches to overcome this. One is Spatially Offset Raman spectroscopy (SORS) as described by Buckley *et al*.^22^ for sub-cutaneous bones like tibia, and the other is a fiber optic probe within a hypodermic needle as described by Petterson *et al*.^46^ for deeper bone analysis, such as hips.

A drawback of this study is that we did not monitor the progression of the osteomyelitis histologically. This would have allowed us to correlate the Raman spectroscopic changes with the histological changes. However, the contribution of the immune response would still be absent in an *in-vitro* study. We are therefore, carrying out an *in-vivo* study, which will include histological examination in order to correlate the histological changes with Raman spectroscopic findings.

From a clinical perspective, use of Raman spectroscopy is likely to add to the armamentarium available to the clinician especially in a condition such as osteomyelitis where early diagnosis and prompt treatment would prevent bone destruction and thereby save the patient from long term morbidity.

## Electronic supplementary material

Supplementary information
