# Supplementary material for: Raman Spectroscopy detects changes in Bone Mineral Quality and Collagen Cross-linkage in Staphylococcus Infected Human Bone
Source: Sci Rep. 2018 Jun 20;8:9417. doi: 10.1038/s41598-018-27752-z (PMC6010429; doi:10.1038/s41598-018-27752-z)
Supplement: Supplementary file 1 — Supplementary information [file 41598_2018_27752_MOESM1_ESM.pdf]

Supplementary Information

Raman Spectroscopy detects changes in Bone Mineral Quality and Collagen Cross-linkage in Staphylococcus Infected Human Bone

Mohamed Khalid[1], Tanujjal Bora[2], Ahmed Al Ghaithi[3], Sharanjit Thukral[4] and Joydeep Dutta[5]*

[1]Department of Orthopaedics, College of Medicine, Taibah University, Universities Road, Taibah, Madinah Al-Munawwarah, 42353, Saudi Arabia

[2]Centre of Excellence in Nanotechnology, Asian Institute of Technology, PO Box 4, Klong Luang, Pathumthani – 12120, Thailand

[3]Oman Medical Specialty Board, Orthopaedic Residency Program, Al-Khoud, Al-Athiba, Oman

[4] Microbiology Department, College of Medicine, Sultan Qaboos University, Al-Khoud - 123, Oman

[5] Functional Materials, Department of Applied Physics, SCI School, KTH Royal Institute of Technology, SE-164 40, Kista, Stockholm, Sweden

[*]**Corresponding author:** joydeep@kth.se, Tel: +46-8-790 81 42

Table T1. A chronological summary of key investigations in the field of Raman Spectroscopy, pertaining to diagnosis of human bone diseases.

Publication year	Authors	Disease	Summary of key findings
2006	McCreadie BR, Morris MD, Chen T-c, Rao DS, Finney WF, Widjaja E et al.[14]	Osteoporosis	Bone adjacent to the fracture less mineralized but with a higher carbonate content compared to bone 2 mm away from the fracture in females with femoral head fractures. Carbonate/amide I ratio significant differentiator between undamaged bone of fractured specimens and undamaged controls.

2011	Gamsjaeger S, Buchinger B, Zoehrer R, Phipps R, Klaushofer K et al.[15]	Osteoporosis - Treatment	Effect of treatment with teriparatide on bone compositional properties in osteoporotic patients was studied by Raman and Fourier Transform Infrared Imaging (FTIR). Significant differences in mineral/matrix, mineral maturity/crystallinity, and collagen cross-link ratio bone quality indices after treatment were observed, indicating a specific response of these patients to the treatment.
2013	Esmonde-White KA, Esmond-White FW, Holmes CM, Morris MD, Roessler BJ.[16]	Diabetic Osteomyelitis	Compositional analysis of diabetic osteomyelitis patients requiring surgical intervention revealed abnormal calcium phosphate minerals including dicalcium phosphate dihydrate (brushite) and uncarbonated apatite.
2013	Gamsjaeger S, Hofstetter B, Zwettler E, Recker R, Gasser JA, Eriksen EF et al.[17]	Osteoporosis - Treatment	Once a year administration of Zoledronic acid (ZOL) for 3 years had a beneficial effect on the progression of the mineral-to-matrix ratio and mineral maturity bone quality indices as evidenced by Raman spectroscopy of iliac crest biopsies of patients on ZOL compared to the placebo group
2013	Busse B, Bale HA, Zimmermann EA, Panganiban B, Barth HD,	Osteomalacia (Vitamin D deficiency in adults)	The hallmark of osteomalacia is an excessive amount of unminer-<br>alized collagen matrix (osteoid)<br>Raman spectroscopy of iliac crest specimens from humans with osteomalacia revealed changes in the amide I band of collagen, changes in the carbonate to

	Carriero A et al.[18]		phosphate ratio, acid phosphate content and crystallinity consistent with a maturing mineralization pattern (ageing).
2014	Olejnik C, Falgayrac G, During A, Vieillard MH, Maes JM, Cortet B et al.[19]	Osteonecrosis (ON)	ON of the jaw secondary to bisphosphonate use was studied. Raman spectroscopy revealed a significant increase of mineral to organic ratio and a significant decrease of relative proteoglycan content. Structural changes on mineral components as revealed by a significant decrease of both crystallinity and mineral maturation was evident compared to healthy bones.
2014	Kim G, Cole JH, Boskey AL, Baker SP, van der Meulen MCH.[20]	Osteoporosis	Cancellous bone from vertebrae was studied. Lower mineral:matrix ratio, crystallinity and carbonate substitution were observed in osteoporotic specimens compared to non-osteoporotic ones.
2014	Imbert L, Auregan J-C, Pernelle K, Hoc T.[21]	Osteogenesis imperfecta (OI)	Raman spectroscopy of samples obtained from children with OI compared to normal controls showed that the mineral-to-matrix ratio was higher in the OI samples, while the crystallinity was lower, suggesting that the mineral crystals were smaller but more abundant in the case of OI.
2014	Buckley K, Kernes JG, Gikas PD, Birch HL, Veniton J, et al.[22]	Osteogenesis imperfecta (OI)	Non-invasive Spatially Offset Raman Spectroscopy (SORS) of a OI patient revealed a significantly more mineralization compared with non-OI control bone; specifically, the phosphate n1 to Amide III ratio was higher. No sample preparation was needed. The laser was

			simply directed through the skin onto fresh bone samples.
2016	Beattie, James R., Niamh M. Cummins, Clare Caraher, Olive M. O'Driscoll, Aruna T. Bansal, et al.[23]	Risk of sustaining fragility fractures	Raman spectroscopy of finger nails in humans was used to predict fragility fracture risk. There were measurable changes at all levels of protein structure of keratin in the fracture group compared with the subjects in the nonfracture group.

References from the main manuscript:

14. McCreadie BR, Morris MD, Chen T-c, Rao DS, Finney WF, Widjaja E et al. Bone tissue compositional differences in women with and without osteoporotic fracture. *Bone*. 39:1190–1195(2006).

15. Gamsjaeger S, Buchinger B, Zoehrer R, Phipps R, Klaushofer K, Paschalis EP. Effects of one-year daily teriparatide treatment on trabecular bone material properties in postmenopausal osteoporotic women previously treated with alendronate or risedronate. *Bone*. 49: 1160–1165 (2011).

16. Esmonde-White, K. A., Esmonde-White, F. W., Holmes, C. M., Morris, M. D., & Roessler, B. J. Alterations to bone mineral composition as an early indication of osteomyelitis in the diabetic foot. *Diabetes care*, *36*(11), 3652-3654 (2013).

17. Gamsjaeger S, Hofstetter B, Zwettler E, Recker R, Gasser JA, Eriksen EF et al. Effects of 3 years treatment with once-yearly zoledronic acid on the kinetics of bone matrix maturation in osteoporotic patients. *Osteoporos Int*. 24:339–347 (2013).

18. Busse B, Bale HA, Zimmermann EA, Panganiban B, Barth HD, Carriero A et al. Vitamin D deficiency induces early signs of aging in human bone, increasing the risk of fracture. *Sci Transl Med*. 5:193ra8 (2013).

19. Olejnik C, Falgayrac G, During A, Vieillard MH, Maes JM, Cortet B et al. Molecular alterations of bone quality in sequesters of bisphosphonates-related osteonecrosis of the jaws. *Osteoporos Int*. 25:747–756 (2014).

20. Kim G, Cole JH, Boskey AL, Baker SP, van der Meulen MCH. Reduced tissue-level stiffness and mineralization in osteoporotic cancellous bone. *Calcif Tissue Int*. 95:125–131 (2014).

21. Imbert L, Auregan J-C, Pernelle K, Hoc T. Mechanical and mineral properties of osteogenesis imperfecta human bones at the tissue level. *Bone*. 65:18–24 (2014).

22. Buckley, Kevin, Jemma G. Kerns, Panagiotis D. Gikas, Helen L. Birch, Jacqueline Vinton, Richard Keen, Anthony W. Parker, Pavel Matousek, and Allen E. Goodship. "Measurement of abnormal bone composition in vivo using noninvasive Raman spectroscopy." *IBMS BoneKEy* 11 (2014).

23. Beattie, James R., et al. "Raman Spectroscopic Analysis of Fingernail Clippings Can Help Differentiate Between Postmenopausal Women Who Have and Have Not Suffered a Fracture." Clinical medicine insights. Arthritis and musculoskeletal disorders 9 .109 (2016).
